# How to Turn a Poisonous Plant into Medicine: Non-Polar Extracts of *Rhododendron adamsii* (Sagan Dalya) Are Free of Grayanotoxins and Inhibit the SARS-CoV-2 Main Protease

**DOI:** 10.3390/molecules31122090

**Published:** 2026-06-14

**Authors:** Tatiana P. Kukina, Ivan A. Elshin, Ol’ga I. Sal’nikova, Svetlana V. Belenkaya, Evgeniia A. Kolosova, Ekaterina A. Volosnikova, Victoria O. Shchegolkova, Dmitry N. Shcherbakov

**Affiliations:** 1 Vorozhtsov Novosibirsk Institute of Organic Chemistry, Siberian Branch of the Russian Academy of Sciences, 630090 Novosibirsk, Russia; olga@nioch.nsc.ru (O.I.S.); belenkaya.sveta@gmail.com (S.V.B.); dnshcherbakov@gmail.com (D.N.S.); 2 Department of Natural Sciences, Novosibirsk State University, 630090 Novosibirsk, Russia; lanosterol@yandex.ru; 3 State Research Center of Virology and Biotechnology VECTOR, Rospotrebnadzor, 630559 Novosibirsk, Russia; kurchanovaea@gmail.com (E.A.K.); volosnikova_ea@vector.nsc.ru (E.A.V.); v.shhegolkova1@mail.ru (V.O.S.); 4 Center for Recombinant Technologies, Altay State University, 656049 Barnaul, Russia

**Keywords:** *Rhododendron adamsii*, grayanotoxins, ursolic acid, oleanolic acid, triterpenic acids, 3CL protease, SARS-CoV-2, GC-MS

## Abstract

The composition of low-polarity extracts obtained by sequential extraction of the aerial parts of *Rhododendron adamsii* Rehd. with hexane and methyl tert-butyl ether (MTBE) was investigated using GC-MS. The hexane extract was dominated by non-polar components: squalene, n-alkanes (nonacosane, hentriacontane), sesquiterpenes (*trans*-nerolidol, spathulenol, β-farnesene), and β-sitosterol. The subsequent MTBE extract was enriched in more polar lipids, primarily free triterpenic acids (ursolic and oleanolic acids). A critical finding was the complete absence of diterpene grayanotoxins in all tested extracts, confirming the safety of the non-polar extraction approach. In bioactivity assays, the total hexane extract demonstrated potent inhibitory activity against the SARS-CoV-2 main protease (3CLpro) with IC_50_ values of 0.0125–0.025 mg/mL, only one order of magnitude higher than the reference inhibitor disulfiram. Fractionation revealed that the activity was distributed among free acids, bound acids, and the unsaponifiable residue, indicating a multicomponent mechanism. Importantly, none of the samples inhibited HIV-1 protease (IC_50_ > 0.1 mg/mL), demonstrating selectivity for the cysteine protease 3CLpro over the aspartyl protease of HIV-1. These results highlight that sequential non-polar extraction of *R. adamsii* provides a grayanotoxin-free lipophilic complex with selective anti-SARS-CoV-2 protease activity, paving the way for bioactivity-guided identification of individual inhibitors.

## 1. Introduction

Medicinal plants that enhance mental and physical performance have traditionally attracted the attention of researchers. Among them, a special place is occupied by representatives of the Ericaceae family, in particular various species of the genus *Rhododendron*, which are used in folk medicine as adaptogenic and tonic agents [1,2].

One of the promising species is *Rhododendron adamsii* Rehd., which grows in the mountainous regions of Eastern Siberia and Mongolia. Previously, we have shown that the ether extract of this plant exhibits inhibitory activity against the main protease (3CLpro) of SARS-CoV-2 [3]. Various extracts of *R. adamsii* have been shown to contain mono- and sesquiterpenes [4], fatty and triterpenic acids [5,6,7,8], sterols and triterpenoids [8], prenylated phenols, and flavonoids [9,10]. Using supercritical CO_2_ extraction and chromatographic–mass spectrometric analysis, more than 50 low-polarity and 170 polar metabolites have been characterized [11,12]. According to a review [13], a total of 208 different metabolites have been identified in plants of the genus *Rhododendron*, including mono-, sesqui-, di-, and triterpenes; steroids; coumarins; iridoids; and other compounds.

In our previous work, exhaustive extraction of *R. adamsii* with methyl tert-butyl ether (MTBE) followed by fractionation into free and bound acids led to the identification of more than 150 triterpenic and aliphatic components [3].

However, despite the obvious interest in *R. adamsii* as a source of biologically active compounds, many researchers overlook an important circumstance. Various *Rhododendron* species (and other ericaceous plants) contain diterpene neurotoxins—grayanotoxins. More than 50 years ago, one of these toxins was isolated from *R. adamsii* [14]. To date, more than 150 diterpene toxins of this class are known, a significant part of which have been found in Ericaceae representatives [15].

The danger is not limited to the consumption of plant parts or herbal teas derived from them, but also includes honey collected by bees during mass flowering of rhododendrons. As noted in [16], diterpene grayanotoxins cause characteristic symptoms of intoxication: dizziness, hypotension, and atrioventricular block. The mechanism of toxic action is associated with irreversible activation of neuronal sodium channels, leading to increased vagal tone. At the same time, the literature contains almost no rigorous evidence of the benefits of grayanotoxin-containing preparations, whereas their potential harm is well documented [16]. A critical review [17] also analyzes in detail both the beneficial and harmful effects of *Rhododendron* extracts and pure grayanotoxins.

Thus, the relevant task arises of separating the beneficial and toxic components of *R. adamsii*. Since grayanotoxins are polar compounds (readily soluble in water and alcohols) [16], it can be assumed that they should not transfer into non-polar (lipophilic) extracts obtained with hydrocarbon solvents.

The aim of this work was to investigate the composition of low-polarity extracts from the aerial parts of *R. adamsii* obtained by sequential extraction with hexane and MTBE, with an emphasis on the absence of grayanotoxins, as well as to evaluate their inhibitory activity against the main proteases of SARS-CoV-2 and HIV-1.

## 2. Results

### 2.1. Yield of Extracts and Fractions

The yield of total lipophilic extracts obtained by sequential extraction of the aerial parts of *Rhododendron adamsii* Rehd. was 13.3% for hexane and 9.4% for the subsequent extraction with methyl tert-butyl ether based on the weight of air-dried plant material. The combined yield of the two sequential extractions exceeded the yield of exhaustive extraction with MTBE alone, which was described previously [3].

Fractionation of the extracts into free acids (FAs), bound acids (BAs), and unsaponifiable residue (UsR) showed that the distribution of components strongly depended on the solvent used (Table 1). The hexane extract was dominated by the UsR fraction (6.1% of the raw material weight), whereas in the MTBE extract the proportion of free acids was higher (6.2%).

### 2.2. Composition of the Unsaponifiable Residue

The unsaponifiable residues of both extracts contained aliphatic hydrocarbons, aldehydes, ketones, alcohols, as well as terpene and phenolic compounds.

#### 2.2.1. Aliphatic Hydrocarbons

In the hexane extract, squalene dominated (323.6 mg/100 g of raw material), whereas in the MTBE extract its content was almost 40 times lower (7.9 mg/100 g). Among the n-alkanes, nonacosane predominated in both extracts (190.9 and 125.2 mg/100 g, respectively). Hentriacontane, tricosane, and pentacosane were also detected (Table 2). The MTBE extract was characterized by the presence of 2-methyltricosane, which was absent from the hexane extract.

#### 2.2.2. Aliphatic Aldehydes and Ketones

In the hexane extract, the following ketones were identified: 2-pentacosanone (5.5 mg/100 g), 2-heptacosanone (12.9 mg/100 g), and 2-nonacosanone (5.5 mg/100 g). The ketone composition of the MTBE extract was significantly richer: 2-heneicosanone, 2-tricosanone, 2-hexacosanone, 2-octacosanone, and 2-hentriacontanone, and geranylacetone and hexahydrofarnesylacetone were additionally detected (Table 3). The content of most ketones was higher in the MTBE extract than in the hexane extract.

#### 2.2.3. Aliphatic Alcohols

In the hexane extract, a wide range of aliphatic alcohols from C16 to C30 were identified, among which 1-docosanol (111.1 mg/100 g) and 1-tetracosanol (119.7 mg/100 g) predominated. The MTBE extract had a less diverse set of alcohols; however, the contents of phytol (20.5 mg/100 g) and 1-eicosanol (20.3 mg/100 g) remained significant (Table 4).

#### 2.2.4. Terpene and Phenolic Components

The fraction of terpenes and phenols turned out to be the richest in composition (Table 5). In the hexane extract, the dominant compounds were *trans*-nerolidol (531.7 mg/100 g), β-sitosterol (353.7 mg/100 g), glutinol (291.0 mg/100 g), spathulenol (253.6 mg/100 g), and β-farnesene (238.2 mg/100 g). In the MTBE extract, conversely, α-amyrin (252.8 mg/100 g) and β-amyrin (52.1 mg/100 g) predominated, while the content of most sesquiterpenes was significantly lower. Notably, the MTBE extract contained β-amyron, β-amyrin, lupeol, cycloartenol, ferninol, and betulin, which were practically not extracted by hexane.

Importantly, no diterpene toxins (grayanotoxins) were detected in any of the samples studied (neither in the total extracts nor in the UR fractions). This finding is consistent with the assumption that the inherently polar grayanotoxins do not partition into non-polar extracts obtained with hydrocarbon solvents.

### 2.3. Composition of Free and Bound Acids

#### 2.3.1. Free Acids

In the free acid fraction of the hexane extract, fatty acids predominated: oleic (193.7 mg/100 g), linoleic (167.6 mg/100 g), lignoceric (156.1 mg/100 g), palmitic (119.4 mg/100 g), and behenic (101.3 mg/100 g). In the MTBE extract, in addition to fatty acids (oleic 172.9 mg/100 g; linoleic 131.5 mg/100 g), high concentrations of pentacyclic triterpenic acids were found: ursolic acid (2596.5 mg/100 g), oleanolic acid (694.2 mg/100 g), as well as ursonic and oleanonic acids. As expected, free triterpenic acids are poorly extracted with hexane due to their low solubility; an exception is the native acetates of ursolic and oleanolic acids, which are less polar (Table 6).

#### 2.3.2. Bound Acids

The composition of bound acids differed from the profile of free acids both in the set of compounds and in their quantitative content (Table 7). In the hexane extract, the bound acid fraction was dominated by oleic (531.6 mg/100 g), linoleic (446.2 mg/100 g), lignoceric (210.8 mg/100 g), and behenic (136.5 mg/100 g) acids, as well as methylated branched-chain derivatives. In the MTBE extract, in addition to fatty acids, ursolic (135.9 mg/100 g) and oleanolic (35.8 mg/100 g) acids were also identified among the bound acids, accounting for approximately 5% of their content in the free acid fraction.

### 2.4. Inhibitory Activity Against Viral Proteases

All tested samples (total extracts and individual fractions) exhibited inhibitory activity against the recombinant main protease of SARS-CoV-2 (Table 8). The highest activity was shown by the total hexane extract, for which the IC_50_ was in the range of 0.0125–0.025 mg/mL (units of measurement are consistent with the control, nirmatrelvir). The free acids, bound acids, and unsaponifiable residue fractions of the hexane extract were characterized by IC_50_ values ranging from 0.021 to 0.044 mg/mL.

The purity of the recombinant SARS-CoV-2 main protease used in these assays was confirmed by SDS-PAGE (Supplementary Figure S1).

Methylation of the free acids led to a certain decrease in activity (IC_50_ increased to 0.036–0.05 mg/mL). Fractions isolated by column chromatography showed IC_50_ values in the range of 0.024–0.03 mg/mL.

Analysis of the chromatographic mass spectra of individual fractions revealed the presence of compounds with nearly identical mass spectra, which may have indicated the presence of isomeric triterpene structures. It is possible that these components made a major contribution to the antiviral activity of the tested fractions.

To assess the selectivity of the inhibitory action, the same extracts and fractions were tested against recombinant HIV-1 protease. The testing protocol (FRET assay using a fluorophore/quencher peptide substrate) was analogous to that described for SARS-CoV-2 3CLpro, with the peptide substrate replaced by one specific for HIV-1 protease.

None of the tested samples showed inhibitory activity within the concentration range studied (up to 0.1 mg/mL). The IC_50_ values for all samples exceeded the maximum tested concentration. This means that the observed inhibition of the SARS-CoV-2 main protease is not a consequence of non-specific protein denaturation or the formation of insoluble complexes with the substrate, but rather has a selective nature.

## 3. Discussion

### 3.1. Effect of Solvent Nature on the Composition of Extracts

In the present work, a systematic comparison of the composition and antiviral activity of sequential non-polar extracts (hexane, then MTBE) from the aerial parts of *Rhododendron adamsii* Rehd. was performed for the first time. The obtained data demonstrate significant differences in the extraction efficiency of the two organic solvents.

Hexane, being a non-polar hydrocarbon, predominantly extracts non-polar components: squalene, aliphatic hydrocarbons (nonacosane, hentriacontane), sesquiterpenes (*trans*-nerolidol, spathulenol, β-farnesene), and sterols (β-sitosterol). In contrast, MTBE, which has a higher polarity and donor ability via its oxygen atom, more effectively extracts polar lipids: free fatty acids, as well as triterpenic acids (ursolic and oleanolic). This result is consistent with known data indicating that MTBE is a more universal extractant for secondary metabolites of medium polarity compared to hexane [3].

The differential extraction of triterpene alcohols deserves special attention. α-Amyrin, β-amyrin, lupeol, and betulin are present predominantly in the MTBE extract, whereas glutinol and β-sitosterol are better extracted with hexane. This is likely due to the different conformations of the hydroxyl groups and the degree of their shielding by the hydrocarbon skeleton. The total yield of the two sequential extractions (hexane + MTBE) exceeds the yield of exhaustive extraction with MTBE alone [3], making the proposed scheme preferable for the most complete recovery of lipophilic components.

### 3.2. Absence of Grayanotoxins as a Key Safety Factor

The most important result of this work is the complete absence of diterpene toxins (grayanotoxins) in all investigated non-polar extracts. Grayanotoxins are polyhydroxylated isoprenoids with high solubility in water and lower alcohols but not in hydrocarbons [16]. Their toxic mechanism is associated with irreversible activation of neuronal sodium channels, leading to hypotension, bradycardia, and atrioventricular block [16]. Although fatal poisonings are rare in humans, they are well documented in cattle and domestic animals [15,17].

Despite the fact that some authors [18] continue to study aqueous–alcoholic extracts of *R. adamsii* as promising adaptogens, the lack of control over grayanotoxin content in such extracts poses a potential danger. Our approach—using sequential non-polar extraction—completely excludes the transfer of these toxins from the raw material into the extract. This makes further studies on cell models and in vivo ethically justified and toxicologically safe.

### 3.3. Inhibition of SARS-CoV-2 Main Protease

The total hexane extract demonstrated the most pronounced inhibitory activity against the SARS-CoV-2 main protease 3CLpro (IC_50_ = 0.0125–0.025 mg/mL). For comparison, the reference inhibitor disulfiram showed an IC_50_ of 0.002 ± 0.0007 mg/mL. The activity of the natural extract is only one order of magnitude lower than that of the synthetic control, which for a complex multicomponent mixture is a remarkably high value.

Fractionation revealed that the activity was distributed among free acids (IC_50_ = 0.044 mg/mL), bound acids (IC_50_ = 0.025 mg/mL), and the unsaponifiable residue (IC_50_ = 0.038 mg/mL). This indicates a multicomponent mechanism of inhibition. The effect appears to be due to the additive or synergistic action of several classes of compounds: triterpenic acids (ursolic and oleanolic), sterols (β-sitosterol, glutinol), and sesquiterpene alcohols (*trans*-nerolidol, spathulenol). Inhibition of 3CLpro by ursolic acid has been reported previously in the literature [3,19]. However, in our extracts, in addition to ursolic acid, many other components are present that may enhance the effect.

Methylation of free acids led to a decrease in activity: for fraction 2 of methylated free acids, the IC_50_ increased from 0.021 to 0.05 mg/mL, and for fraction 3, from 0.025 to 0.036 mg/mL. This indirectly confirms that the free carboxyl group is important for binding to the active site of the protease, presumably through the formation of hydrogen bonds with the catalytic residues Cys145 and His41.

Our results are consistent with recent findings on another *Rhododendron* species. Lingwan et al. (2023) demonstrated that a hot aqueous extract of *Rhododendron arboreum* petals inhibits SARS-CoV-2 infection in Vero E6 cells (IC_50_ = 173 μg/mL) and that its major phytochemicals, such as 5-O-feruloylquinic acid and caffeoylquinic acids, exhibit binding affinity to 3CLpro in molecular docking studies [20]. Although the extraction methods and chemical profiles differ (non-polar extracts of *R. adamsii* rich in triterpenic acids vs. aqueous extracts of *R. arboreum* rich in quinic acid derivatives), both studies independently point to the *Rhododendron* genus as a promising source of natural 3CLpro inhibitors.

### 3.4. Selectivity of Inhibition: Lack of Activity Against HIV-1 Protease

An important finding that confirms the specificity of the observed effect is the complete absence of inhibitory activity of all tested samples against HIV-1 protease (Table 8). Whereas inhibition of SARS-CoV-2 3CLpro was observed in the IC_50_ range of 0.0125–0.1 mg/mL, for HIV-1 protease the IC_50_ values exceeded the maximum tested concentration (0.1 mg/mL), and inhibition at this concentration did not reach 5%. The positive control (indinavir) demonstrated expected high activity (IC_50_ ~0.05 µM), confirming the functionality of the enzymatic system.

This difference can be explained by several factors. First, structural differences in the active sites play a key role. HIV-1 protease belongs to the class of aspartyl proteases (its active site contains Asp25/25′), whereas SARS-CoV-2 3CLpro is a cysteine protease with a catalytic dyad Cys145-His41. The compounds identified in the extracts (ursolic and oleanolic acids, β-sitosterol, sesquiterpenes) are capable of covalent or non-covalent interactions with the thiol group of cysteine but do not bind to the aspartate carboxylate.

Second, the well-documented selectivity of triterpenic acids should be noted. It has been repeatedly reported in the literature that ursolic and oleanolic acids exhibit inhibitory activity primarily against cysteine proteases (including 3CLpro and papain-like proteases of coronaviruses) but do not affect serine or aspartyl proteases at comparable concentrations.

Third, the absence of activity against HIV-1 protease allows us to reject the hypothesis that 3CLpro inhibition is associated with simple protein denaturation or protein aggregation in the presence of lipophilic components of the extracts. If such non-specific effects were occurring, they would be observed for both proteases, since they were tested under similar buffer conditions and using the same protocol.

Thus, the obtained data indicate that *R. adamsii* extracts are selective inhibitors of the cysteine protease 3CLpro of SARS-CoV-2 and do not affect the aspartyl protease of HIV-1. This increases the attractiveness of these extracts as a basis for further development of specific antiviral agents.

### 3.5. Limitations of the Study and Future Perspectives

Despite the promising results, this work has a number of limitations that should be considered when interpreting the data.

First, the IC_50_ values were only determined in a cell-free system (FRET assay with recombinant enzymes). There are no data on the cytotoxicity of the extracts on mammalian cell cultures (e.g., Vero, HEK293, and Calu-3) or on the selectivity index (CC_50_/IC_50_). Without these data, it is impossible to assess the therapeutic potential of the extracts in vivo.

Second, the absence of tests on live viruses should be noted. Inhibition of a recombinant protease does not guarantee antiviral activity in cell culture, since extracts may fail to penetrate cells, may be metabolized, or may bind to serum proteins.

Third, the mechanism of inhibition has not been characterized. It is unknown whether the inhibition is competitive, non-competitive, or mixed. To answer this question, Michaelis–Menten kinetic studies at different inhibitor concentrations are required.

Fourth, the identification of the active components is not yet complete. Although we assume a contribution from ursolic acid, β-sitosterol, and sesquiterpenes, direct proof of their role requires the isolation of each compound in individual form and testing in the same FRET assay, followed by comparison with the activity of the original mixture (bioactivity-guided fractionation).

Addressing these limitations in future studies will allow a more complete understanding of the antiviral potential of *R. adamsii* extracts and will bring us closer to the development of standardized phytopreparations with proven efficacy and safety.

## 4. Materials and Methods

### 4.1. Plant Material

The aerial parts of *Rhododendron adamsii* Rehd. were collected at the post-flowering stage (August 2023) in the vicinity of Orlik village, Republic of Buryatia, Russia. Whole, healthy, undamaged plants were collected, cleaned from dust if necessary, and air-dried at room temperature indoors with no exposure to direct sunlight. The authenticity of the raw material was confirmed by D. N. Shcherbakov, PhD in Biology.

### 4.2. Preparation of Rhododendron adamsii Extracts

The air-dried raw material was ground in a screw crusher and sieved through a 2 mm mesh. A weighed portion (100 g) was loaded into a Soxhlet extractor and extracted with hexane for 20 h (3 × 6–7 h). After extraction with hexane, the raw material was extracted with methyl tert-butyl ether (MTBE) (PanReac AppliChem, Darmstadt, Germany) for another 20 h (3 × 6–7 h) without unloading from the thimble (sequential extraction). The yields were 13.3% for the hexane extract and 9.4% for the MTBE extract (wt% of the raw material mass).

### 4.3. Sample Preparation for GC-MS Analysis

Sample preparation for GC-MS analysis included isolation of free acids with an alkaline extractant (2% aqueous NaOH solution) and hydrolysis of the extract after removal of free acids using a 15% water–ethanol solution of KOH. The solution of the sodium salts of free acids was acidified with 10% HCl, and free acids were extracted four times with MTBE and washed with distilled water to obtain a neutral reaction. The product of alkaline hydrolysis was four-fold diluted with water and extracted four times with MTBE; the MTBE extract was then washed with distilled water to achieve a neutral reaction. The aqueous solution of the potassium salts of bound acids was acidified with 10% HCl, and the acids were extracted four times with MTBE and washed with distilled water to achieve a neutral reaction. From each extract, three fractions were obtained: free acids, bound acids, and unsaponifiable residue (UR). The acid components were methylated with diazomethane, while neutral components were analyzed without derivatization. The neutral substances of the UR were subjected to chromatographic separation on a silica gel column (60–200 μm, Merck, Darmstadt, Germany) using hexane as the eluent with diethyl ether content increasing from 0 to 50% (by volume). Fractions of 12–15 mL were collected. Fractions were combined according to the results of thin-layer chromatography on Sorbfil and Silufol plates (Sorbpolymer, Krasnodar, Russia). The chromatograms were developed using a mixture of hexane with MTBE in a concentration from 10 to 50% (by volume). Detection was carried out with a mixture of vanillin with sulfuric acid and ethanol in a ratio of 1:10:90 (by mass), followed by heating. As a result, concentrates of hydrocarbons, ketones, and aliphatic and terpene alcohols (including sterols and diols) were obtained and analyzed by GC-MS at the Multi-Access Chemical Research Centre, Siberian Branch of the Russian Academy of Sciences.

### 4.4. GC-MS Analysis

GC-MS analysis was performed on an Agilent Technologies instrument (Santa Clara, CA, USA) consisting of an Agilent 6890N gas chromatograph and an Agilent 5973N mass-selective detector (EI, 70 eV) using an HP-5MS capillary column (5% diphenyl/95% dimethylsiloxane; 30 m × 0.25 mm × 0.25 μm film thickness). The analysis parameters were as follows: helium carrier gas at 1 mL/min; oven temperature program: 50 °C for 2 min, then ramp 10 °C/min to 300 °C and hold at 300 °C for 30 min; injector temperature 280 °C; ion source temperature 170 °C; and scan rate 2.4 scans/s in the mass range *m*/*z* 30–650. Components were identified using the NIST2020 Library of Mass Spectral Data (more than 500,000 compounds) and the literature [19]. The contents (mg/100 g of raw material) were determined from peak areas without the use of correction coefficients; the percentage of correspondence between individual components and the database was within 75–90%.

### 4.5. Production of Recombinant 3CLpro and HIV-1 Proteases and Determination of Inhibitory Activity

Recombinant SARS-CoV-2 main protease (3CLpro) was produced by expression in *E. coli* cells followed by purification using metal-affinity chromatography on Ni-Sepharose, as described previously [3]. Expression was induced with IPTG. Protein purity was monitored by SDS-PAGE. Protein concentration was determined by the Bradford method.

Compound activity was assessed by inhibition of cleavage of the fluorescent peptide substrate Dabcyl-KTSAVLQ↓SGFRKME(Edans)-NH_2_ (purity > 95%, CPC Scientific Inc., Hangzhou, China), which contains the 3CLpro recognition site. The principle of the method is based on FRET: upon substrate cleavage by the protease, the fluorophore (Edans) and quencher (Dabcyl) are separated, leading to an increase in fluorescence.

Fluorescence was recorded on a CLARIOstar Plus microplate fluorimeter–spectrophotometer (BMG Labtech, Ortenberg, Germany) at excitation and emission wavelengths of 355 nm and 460 nm, respectively. The reaction mixture contained Tris-HCl buffer (pH 7.3) with EDTA and DTT, peptide substrate (10 μM), recombinant 3CLpro (300 nM), and the test inhibitor at various concentrations. To determine IC_50_, the inhibitor concentration was varied from 400 to 0 μM. Disulfiram was used as a positive control. All measurements were performed in triplicate in kinetic scanning mode. Recombinant HIV-1 protease expressed in *E. coli* was used as a negative control. The recombinant plasmid pET28-HIV was designed to express a single open reading frame encoding a SUMO-HIV-1 protease fusion protein. The vector backbone and HIV-1 protease insert were amplified by PCR. The assembled pET28-HIV plasmid was transformed into chemically competent *E. coli* BL21(DE3) cells. Expression and purification of recombinant HIV-1 protease were performed according to established protocols [21,22].

### 4.6. Statistical Analysis

All experiments were performed in at least three independent biological replicates, each comprising two technical replicates (*n* = 3). Data are presented as mean ± standard deviation (SD) or as concentration ranges where appropriate (e.g., for pooled chromatographic fractions). The four-parameter logistic (4PL) regression model was applied to the combined data from all replicates to calculate IC_50_ values and their 95% confidence intervals using GraphPad Prism 9.0 (GraphPad Software, San Diego, CA, USA). For samples consisting of pure compounds or well-defined fractions, IC_50_ uncertainty was expressed as standard errors of the parameter estimates derived from nonlinear regression.

## 5. Conclusions

Sequential extraction of *Rhododendron adamsii* aerial parts with hexane followed by methyl tert-butyl ether yielded complementary lipophilic fractions with distinct metabolic profiles. The hexane extract was enriched in non-polar compounds (squalene, alkanes, sesquiterpenes, β-sitosterol), while the subsequent MTBE extract was dominated by triterpenic acids (ursolic and oleanolic acids). Critically, GC-MS analysis confirmed the complete absence of diterpene grayanotoxins in all non-polar extracts, establishing a key safety advantage over traditional water–alcohol preparations from *Rhododendron* species.

The hexane extract exhibited potent and selective inhibitory activity against the SARS-CoV-2 main protease with IC_50_ values of 0.0125–0.025 mg/mL, only one order of magnitude higher than the reference inhibitor disulfiram. The activity was distributed among free acids, bound acids, and the unsaponifiable residue, suggesting a multicomponent mechanism. None of the samples inhibited HIV-1 protease (IC_50_ > 0.1 mg/mL), demonstrating selectivity for the cysteine protease 3CLpro over the aspartyl protease of HIV-1. These findings provide a safe, grayanotoxin-free lipophilic complex from *R. adamsii* with selective anti-SARS-CoV-2 protease activity, and pave the way for bioactivity-guided identification of individual 3CLpro inhibitors.

## Figures and Tables

**Table 1:** Yields of total extracts and their fractions (free acids, bound acids, and unsaponifiable residue) obtained by sequential extraction of *R. adamsii* aerial parts with hexane followed by MTBE. Values are expressed as percentage of air-dried plant material weight (*w*/*w*%).

Fraction/Extragent	Hexane	MTBE/Hexane
Total extract (TE)	13.3	9.4
Free acids (FAs)	4.3	6.2
Bound acids (BAs)	2.9	1.2
Unsaponified residue (UsR)	6.1	2.00

**Table 2:** Aliphatic hydrocarbons identified in the unsaponifiable residue fractions of hexane and MTBE extracts from *R. adamsii*. Values are expressed as mg/100 g (mg%) dry plant weight.

Constituent	Retention Time	Hexane Extract	MTBE/Hexane Extract
Heneicosane	21.153	2.5	1.6
Docosane	22.055		0.6
Tricosane	22.929	43.0	22.9
2-Methyltricosane	23.456		5.9
Tetracosane	23.759	10.4	3.5
Pentacosane	24.560	20.3	15.0
Hexacosane	25.318	2.5	2.8
Heptacosane	26.069	9.2	8.3
Octacosane	26.791	1.8	3.0
Nonacosane	27.535	190.9	125.2
Triacontane	28.300	4.9	2.0
Hentriacontane	29.217	55.9	30.4
Tritriacontane	31.477	5.5	2.2
Squalene	27.072	323.6	7.9
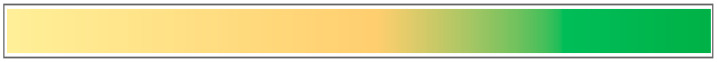			
0.01 mg%	325.0 mg%.		

**Table 3:** Aliphatic aldehydes and ketones identified in the unsaponifiable residue fractions of hexane and MTBE extracts from *R. adamsii*. Values are expressed as mg/100 g (mg%) dry plant weight.

Constituent	Retention Time	Hexane Extract	MTBE/Hexane Extract
Dodecanal			2.2
2-Heneicosanone			0.8
2-Tricosanone			1.4
2-Pentacosanone	26.202	5.5	22.5
2-Hexacosanone	26.935		1.2
2-Heptacosanone	27.679	12.9	26.8
2-Octacosanone	28.184		0.8
2-Nonacosanone	29.455	5.5	6.3
2-Hentriacontanone	31.866	1.1	2.6
Geranylacetone			2.0
Hexahydrofarnesylaceton	18.691		18.3
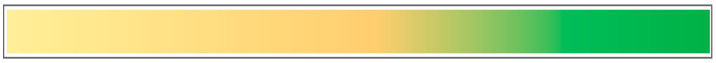			
0.01 mg%	26.8 mg%		

**Table 4:** Aliphatic alcohols identified in the unsaponifiable residue fractions of hexane and MTBE extracts from *R. adamsii*. Values are expressed as mg/100 g (mg%) dry plant weight.

Constituent	Retention Time	Hexane Extract	MTBE/Hexane Extract
1-Hexadecanol	19.037	4.3	
1-Octadecanol	21.023	16.6	4.5
Phytol	21.319	28.2	20.5
1-Nonadecanol	21.622	6.1	
1-Eicosanol	22.835	73.7	20.3
1-Heneicosanol	23.383	38.1	3.7
1-Docosanol	24.517	111.1	38.5
1-Tricosanol	25.658	2.5	
1-Tetracosanol	26.076	119.7	41.0
1-Pentacosanol	26.531	7.6	
1-Hexacosanol	27.549	46.7	15.4
1-Octacosanol	29.289	13.5	3.4
1-Triacontanol	31.664	4.3	1.1
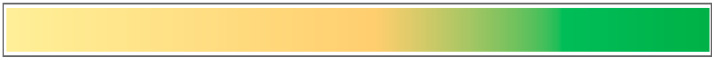			
0.01 mg%	120.0 mg%		

**Table 5:** Terpenoids and phenolic compounds identified in the unsaponifiable residue (UsR) fractions of hexane and MTBE extracts from *R. adamsii*. Values are expressed as mg/100 g (mg%) dry plant weight.

Constituent	Retention Time	Hexane Extract	MTBE/Hexane Extract
3-Phenyl-2-butanone	11.428	8.6	0.4
4-Phenyl-2-butanone	11.435	16.0	1.0
4-Phenylbutanol	11.61	105.0	43.8
(-)-Isoledene	13.310	1.2	
Selina-5,11-diene	13.608	8.3	
α-Gurjunene	13.825	1.2	
Aromadendrene	14.229	76.8	28.6
β-Farnesen	14.279	238.2	14.6
Humulene	14.409	17.8	1.4
(-)-Dehydroaromadendrane	14.452	1.8	
Allo-aromadendrene	14.503	2.5	
γ-Muurolene	14.648	6.1	2.6
γ-Amorphen	14.705	1.8	
Germacrene D	14.727	6.8	
β-Selinene	14.828	8.6	4.9
α-Farnesen	14.879	93.4	9.3
(+)-Ledene	14.922	8.0	1.0
γ-Cadinene	15.131	3.1	1.6
δ-Cadinene	15.218	10.4	0.8
Selina-4(15),7(11)-diene	15.420	79.8	
*trans*-Nerolidol	15.651	531.7	21.9
2-Phenylethanol	15.661	171.9	30.8
Epiglobulol	15.723	17.8	0.6
Spathulenol	15.925	253.6	20.5
Globulol	16.019	50.9	6.1
Caryophyllenoxide	16.020	41.1	
Iso-caryophyllen-β-oxide	16.192	17.2	
β-Elemenone	16.200	65.1	8.5
Humulene-2,3-epoxide	16.207		1.4
Rosifoliol	16.228	5.5	
Humulene-6,7-epoxide	16.337	21.5	19.1
Muurola-4,10(14)-dien-1-β-ol	16.496		2.0
7-Epi-eudesmol	16.539	2.5	
β-Eudesmol	16.728	29.5	6.3
Porosadienol	16.734	15.4	13.2
α-Cadinol	16.799	2.3	
3,5,11-Eudesmatriene	16.813	31.9	
Germacrone	16.979	26.6	22.3
(1*R*,7*S*)-Germacra-4(15),5,10(14)-trien-1β-ol	17.189	7.4	
Germazone	17.203	2.8	46.4
Germacrone (isomer)	17.319	40.3	3.9
γ-Tocopherol	27.065	2.5	
α-Tocopherol (Vitamin E)	29.780	6.1	
Campesterol	31.087	5.8	4.6
Obtusifoliol	32.004	2.1	1.8
Stigmasterol	31.924	12.1	5.7
β-Sitosterol	32.393	353.7	85.0
β-Amyrone	32.567		0.2
Stigmastanol	32.575	0.8	1.4
β-Amyrin	32.971		52.1
α-Amyrone	33.281	2.5	0.4
Cycloartenol	33.498		1.6
Lupeol	33.700		2.3
α-Amyrin	33.866	28.2	252.8
Stigmasta-3,5-dien-7-one	33.780		1.4
Lanosta-8,24-dien-3-one	34.075		1.2
24-Methylencycloartanone	34.220		2.3
24-Methylencycloartanol	34.494		1.8
Fernenol	34.494		14.2
Citrostadienol	34.494		1.2
Glutinol	35.375	291.0	54.5
Friedelin	36.111	83.9	11.2
Eritrodiol	39.209	6.3	15.0
Uvaol	40.530	9.3	20.7
Betulin	41.050	8.6	8.3
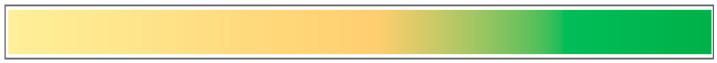			
0.01 mg%	540.0 mg%		

**Table 6:** Free acid composition of hexane and MTBE extracts from *R. adamsii*. Values are expressed as mg/100 g (mg%) dry plant weight.

Constituent		Hexane Extract	MTBE/Hexane Extract
Salicylic		9.8	
3-Phenyllactic		9.5	431.2
Anisic		2.3	
4-Hydroxybenzoic			42.0
Nonandioic			21.5
2-Butanone, 4-(4-hydroxyphenyl)			65.7
10-Undecenoic		8.5	
10-Methylundecanoic		12.2	
Rhododendrol (4-[(3*R*)-3-hydroxybutyl]phenol			25.0
Lauric		2.7	
Vanillic			20.0
Veratric			85.4
Orsellinic			46.4
11-Methyldodecanoic		15.2	
12-Methyltridecanoic		21.5	
3,5,6-Trimethylresorcylic		12.6	21.2
Myristic		9.3	
Undecanedioic		9.3	18.9
2,4-Dihydroxy-3,6-dimethyl-benzoic			28.5
4-(3-Methoxy-3-oxo-1-propenyl-benzoic			44.3
5-Hydroxy-7-methoxy-2-methylchromon			200.9
Palmitic		119.4	85.6
Palmitoleic		9.3	3.2
*cis*-Heptadecenoic		10.2	
Margaric		8.8	62.3
13-Octadecenoic		11.5	
16-Methylheptadecanoic		32.0	
Oleic		193.7	172.9
Linolenic			
Linoleic		167.6	131.5
Stearic		71.5	54.5
Sterculic		29.0	
Arachidic		67.4	35.5
Docosenoic		15.8	
Behenic		101.3	
Tricosanoic		22.0	
Lignoceric		156.1	53.3
Pentacosanoic		26.0	
Cerotic		98.7	
Montanic		66.5	
Melissic		74.3	40.6
Oleanonic			11.2
Ursonic			36.5
Oleanolic			694.2
Ursolic			2596.5
Betulinic			45.6
Acetyloleanolic		30.5	
Acetylursolic		34.8	
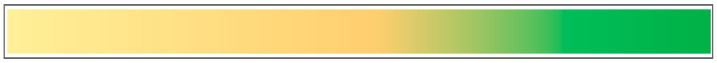			
0.01 mg%	2600.0 mg%		

**Table 7:** Bound acid composition of hexane and MTBE extracts from *R. adamsii*. Values are expressed as mg/100 g (mg%) dry plant weight.

Constituent		Hexane Extract	MTBE/Hexane Extract
Caprilic			2.9
9-Oxononanoic			6.9
9-Oxodecanoic			1.6
Nonandioic			3.2
10-Methylundecanoic		15.2	
Lauric		11.4	4.5
11-Methyldodecanoic		25.7	
12-Methyltridecanoic		37.8	
Tridecanoic			1.8
10-Oxo-8-decenoic			16.4
Miristic		14.8	11.2
13-Methyltetradecanoic		10.4	
Pentadecanoic			1.4
Iso-pentadecanoic			1.7
Palmitic		132.4	80.9
Iso-palmitic		30.7	
Palmitoleic		14.0	1.5
*cis*-Heptadecenoic		18.8	
Margaric		14.4	3.3
15-Methylhexadecanoic		21.4	
Iso-heptadecanoic			3.0
13-Octadecenoic		12.5	
16-Methylheptadecanoic		74.2	
Oleic		531.6	32.7
Linolenic			
Linoleic		446.2	36.9
Stearic		111.6	18.1
Nonadecenoic		22.2	
10-Nonadecenoic		69.0	
17-Methyloctadecanoic		156.8	
Nonadecanoic		13.6	4.5
Dihydrosterculic		48.3	6.1
Hexadecandioic			4.3
18-Methylnonadecanoic		9.2	
13-Eicosenoic		8.6	
Arachinic		82.9	16.9
18-Methyleicosanoic		24.6	
Heneicosanoic		10.1	3.9
Docosenoic		10.5	
Octadecandioic			2.9
Behenic		136.5	27.4
Tricosanoic		11.8	2.8
21-Methyldocosanoic		8.1	
Eicosandioic			5.8
Iso-tricosanoic			1.1
Lignoceric		210.8	27.2
Pentacosanoic			1.7
Iso-pentacosanoic			1.8
Docosandioic			2.0
Cerotinic		53.7	1.1
Montanic		19.8	5.7
Melissic		15.6	14.9
Oleanolic			35.8
Ursolic			135.9
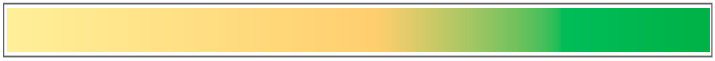			
0.01 mg%	550.0 mg%		

**Table 8:** Values of the half-inhibitory concentration of extracts and fractions from non-polar extracts of *R. adamsii*.

Sample	IC_50_, mg/mL
Hexane extract	0.0125–0.025
Fractions of hexane extract	
Free acids	0.044
Bound acids	0.025
Unsaponifiable residue	0.038
Column chromatography fractions	
Free acids, fraction 2	0.021
Free acids, fraction 5	0.025
Methylated free acids, fraction 2	0.05
Methylated free acids, fraction 3	0.036
Bound acids, fraction 11	0.024
Bound acids, fraction 12	0.03
Reference inhibitor	
Nirmatrelvir	0.0525 ± 0.0045

## Data Availability

The original contributions presented in the study are included in the article; further inquiries can be directed to the corresponding author.

## Supplementary Materials

The following supporting information can be downloaded at: https://www.mdpi.com/article/10.3390/molecules31122090/s1, Figure S1. SDS-PAGE analysis of purified recombinant enzymes.

## Abbreviations

The following abbreviations are used in this manuscript:

3CLpro	Main SARS-CoV-2 protease
GC-MS	Gas chromatography–mass spectrometry
mg%	mg/100 g of raw material
MTBE	Methyl tert-butyl ether
MTBE-extract	Extract obtained with MTBE as extractant
MTBE/Hexane-extract	Extract obtained with MTBE after hexane extraction
RT	Retention time
IC_50_	Half-inhibitory concentration
TE	Total extract
UR	Unsaponifiable residue
