# Supplementary material for: How to Turn a Poisonous Plant into Medicine: Non-Polar Extracts of Rhododendron adamsii (Sagan Dalya) Are Free of Grayanotoxins and Inhibit the SARS-CoV-2 Main Protease
Source: Molecules. 2026 Jun 14;31(12):2090. doi: 10.3390/molecules31122090 (PMC13306085; doi:10.3390/molecules31122090)
Supplement: Supplementary file 1 [file molecules-31-02090-s001.zip › molecules-4330513-supplementary.pdf]

Supplementary Figure S1

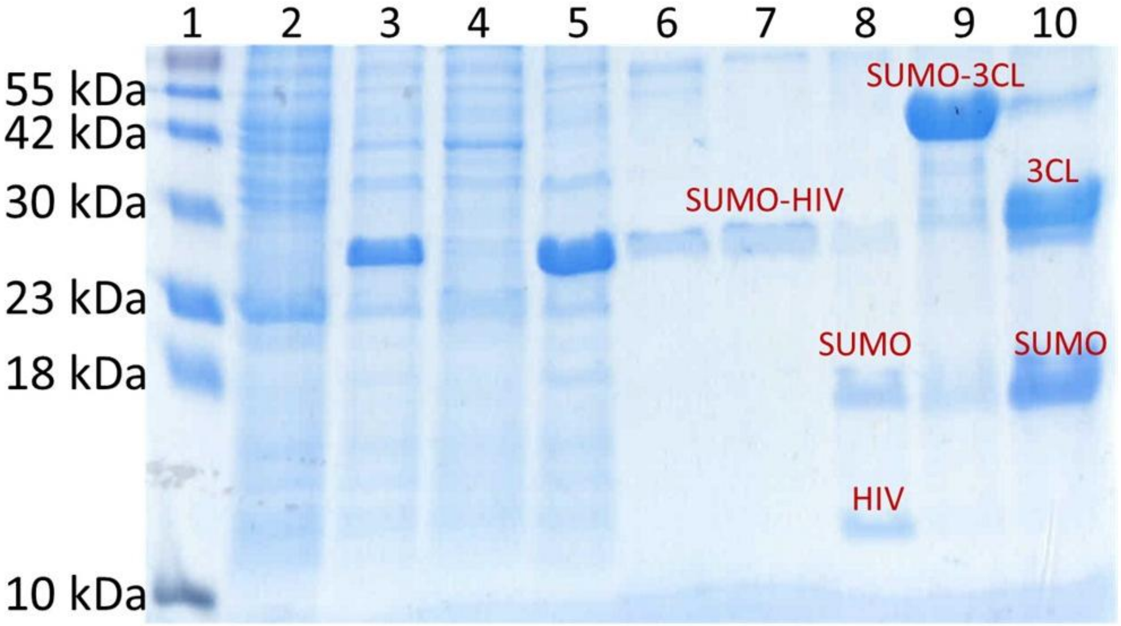

Supplementary Figure S1. SDS-PAGE analysis of purified recombinant enzymes.

1 –Molecular weight marker; 2 – Cell biomass before induction; 3 – Cell biomassafter induction and cultivation; 4 – Soluble cell fraction after induction; 5 –Inclusion bodies after dissolution in urea solution; 6 – Pellet after rapidfolding; 7 – Solution after rapid folding; 8 – Solution after rapid foldingtreated with Ulp protease; 9 – SUMO-3Cl fusion protein; 10 – SUMO-3Cl fusionprotein after Ulp protease treatment.
